# Supplementary material for: Prodigiosin of Serratia marcescens ZPG19 Alters the Gut Microbiota Composition of Kunming Mice
Source: Molecules. 2021 Apr 9;26(8):2156. doi: 10.3390/molecules26082156 (PMC8069934; doi:10.3390/molecules26082156)
Supplement: Supplementary file 1 [file molecules-26-02156-s001.pdf]

Supporting Information

Prodigiosin of *Serratia marcescens* ZPG19 Alters the Gut Microbiota Composition of Kunming Mice

Xue Li [1], Xinfeng Tan [1], Qingshuang Chen [1], Xiaoling Zhu [2], Jing Zhang [1,*], Jie Zhang [1,*] and Baolei Jia [1,*]

[1] School of Bioengineering, State Key Laboratory of Biobased Material and Green Papermaking, Qilu University of Technology (Shandong Academy of Sciences), Jinan,250000 China
[2] Shandong Academy of Agricultural Sciences, Jinan, 250000 China

Table S1. Mouse diet formula.

Formula composition	content（%）
wheat	56.000
Skimmed milk powder	20.000
Soybean meal	11.250
Vegetable oil	5.750
beer yeast	4.000
salt	1.375
Dicalcium Phosphate	1.000
Ferric citrate	0.125
Vitamins	0.500

Table S2. Sequencing results of caecal contents.

Sample code	Raw PE	Effective tags	Effective ratio (%)	Average length
C1	114810	109917	95.74	450
C2	103380	99183	95.94	454
C3	106881	102313	95.73	452
PF1	107929	103270	95.68	455
PF2	107621	102483	95.23	453
PF3	107730	103511	96.08	448

Table S3. Number of OTUs per sample.

Sample code	Tag number	OTU number
C1	109917	937
C2	99183	1002
C3	102313	1059
PF1	103270	1062
PF2	102483	1007
PF3	103511	1350

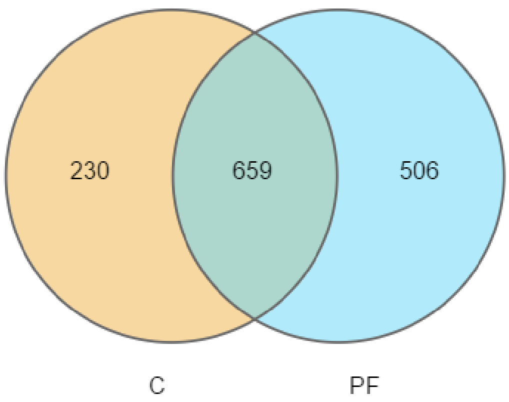

Figure S1. Venn diagram of OTU numbers.

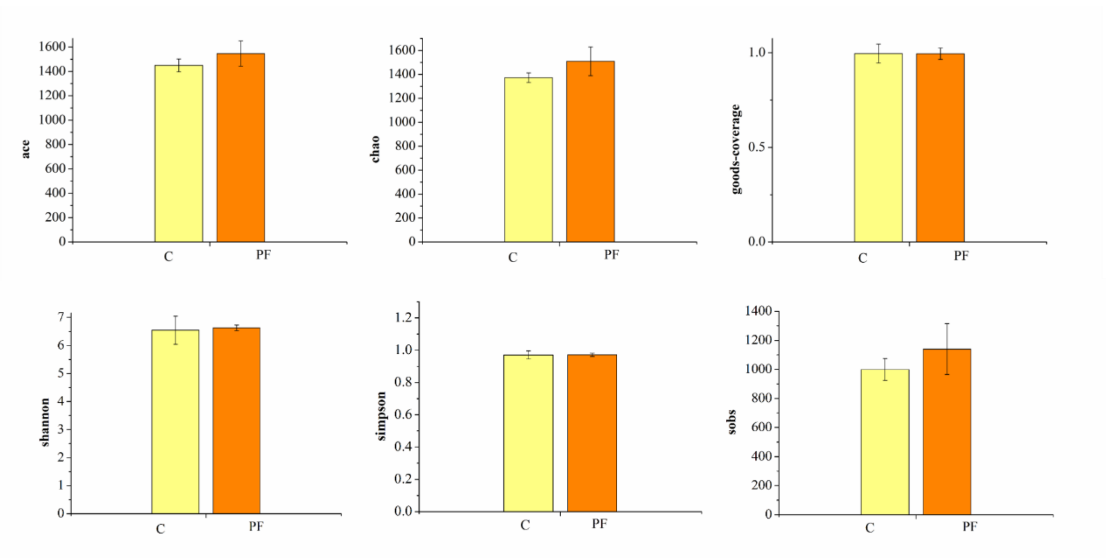

Figure S2. α-Diversity based on the total number of OTUs.
